# Mortality Prediction of Biochemical Parameters in Patients Intubated in the Emergency Department

**DOI:** 10.7759/cureus.73508

**Published:** 2024-11-12

**Authors:** Yavuzselim Koca, Öner Bozan, Meltem Polat, Asim Kalkan

**Affiliations:** 1 Department of Emergency Medicine, Prof. Dr. Cemil Taşcıoğlu City Hospital, Istanbul, TUR

**Keywords:** biochemical parameters, critical care, intubation, lactate, mortality

## Abstract

Introduction

Tracheal intubation is a high-risk airway management protocol frequently applied in patients with critical illnesses. Numerous parameters have been suggested to predict mortality in these patients. Blood gas analysis, electrolyte levels, enzyme activities, and other biochemical measurements provide insights into a patient’s metabolic status and organ functions. Accordingly, it is considered that these parameters have a significant potential for predicting the clinical outcomes of intubated patients. The study aimed to investigate the role of biochemical parameters in determining the 24-hour mortality risk of patients intubated in the emergency department and understand the potential significance of these parameters in predicting the clinical prognosis of these patients.

Methods

The present study was conducted on 1,236 patients who were intubated within a 1.5-year period at the Emergency Medicine Clinic of a tertiary Education and Research Hospital. Lactate, hemoglobin (Hgb), platelets (PLT), pH, HCO_3_, K, urea, creatinine, high-sensitivity troponin I (HS troponin I), and serum sodium levels were recorded for each patient in a data form. The 24-hour mortality rates were then analyzed based on these test results and comorbidities in the patients, and the data were recorded.

Results

The study included 702 patients after reviewing 1,236 cases. The median/mean values of HCO_3_, PLT, and pH were significantly higher in survivors compared to those who did not survive within 24 hours. Conversely, the median/mean values of lactate, creatinine, potassium, and HS troponin I were significantly higher in the patients who lost their lives within 24 hours than in the survivors. Epilepsy status, HCO_3_, lactate, potassium, and PLT values were statistically significant in the multivariate model in predicting 24-hour mortality.

Conclusion

The results of this study indicate that specific laboratory values, particularly blood gas analysis, play a significant role in predicting mortality among patients who present to the emergency department and undergo rapid sequence intubation. Patient prognosis can be predicted using these parameters, and treatment can be planned accordingly. Future multicenter prospective studies using standardized patient-specific intubation could provide further evidence for using the parameters in question in predicting mortality.

## Introduction

The management of the airway, which plays a crucial role in preserving the vital functions of emergency patients, is of paramount importance. Endotracheal intubation stands out as the most frequently employed technique for advanced airway management [1,2]. Tracheal intubation is a commonly used procedure in critically ill patients; however, it is associated with high risks. Patients receiving invasive respiratory support are always exposed to life-threatening risks due to the underlying reasons leading to intubation and the complications that may arise thereafter [3-5]. Shock, respiratory failure, metabolic acidosis, and other pathophysiological changes significantly increase the risk of peri-intubation events in critically ill patients intubated in the emergency department [3,6].

Numerous parameters are considered to predict mortality in patients who undergo intubation due to chronic conditions or acute respiratory failure [7]. Factors such as age, race, time to hospital admission, blood parameters, duration of intensive care unit stay, and reason for intubation provide insight into the survival prospects of patients, assisting emergency medicine specialists in planning treatment [8]. Biochemical parameters are considered a relatively rapid and objective means of assessing the patient’s physiological status [9,10]. Blood gases, electrolyte levels, enzyme activities, and other biochemical measurements reflect the patient’s metabolic and organ functions. Therefore, these parameters hold potential significance in predicting the clinical prognosis of intubated patients. There is an increasing number of studies on the role of these biochemical parameters in determining the severity of illness [7,11,12].

The aim of this study is to investigate the role of biochemical parameters that can be used to determine the 24-hour mortality risk in patients intubated in the emergency department and to better understand the potential significance of these parameters in predicting the clinical prognosis of patients.

## Materials and methods

Study design

This study retrospectively included patients who presented to the Emergency Medicine Clinic of a tertiary education and research hospital within a one-and-a-half-year period and underwent intubation. Ethical approval for this study was obtained from Prof. Dr. Cemil Taşcıoğlu City Hospital Clinical Research Ethics Committee with approval number 350. A total of 1,236 patients were initially enrolled; however, 534 patients were excluded for not meeting the inclusion criteria (aged <18 years, trauma, intubated following a crash injury, diagnosed with COVID-19, and incomplete medical history or routine tests). Ultimately, 702 patients were included in the study.

Following the initial assessment, demographic data (age, gender, and medical history) of eligible patients were recorded, along with levels of lactate, hemoglobin (Hgb), platelets (PLT), pH, HCO_3_, K, urea, creatinine, high-sensitivity troponin I (HS troponin I), and serum sodium. The hospital information system was used to review the test results, additional medical conditions of the patients, and their 24-hour status, and the data were recorded accordingly.

Statistical analyses

Descriptive statistics, including the median, minimum, and maximum values for continuous variables, were tabulated to summarize the study data. Categorical variables were summarized as numbers and percentages.

The normal distribution for numerical variables was tested using Shapiro-Wilk, Kolmogorov-Smirnov, and Anderson-Darling tests. The independent samples t-test was used for normally distributed variables to compare 24-hour mortality data of patients with relevant laboratory parameters, while the Mann-Whitney U test was employed for non-normally distributed variables. Differences in sex and comorbid conditions according to 24-hour mortality data of patients were evaluated using Pearson’s chi-square test.

Receiver operating characteristic (ROC) curve analysis was employed to investigate the performance of certain laboratory parameters (lactate, hemoglobin, platelets, pH, HCO3, K, urea, creatinine, HS troponin I, Na) in predicting 24-hour mortality. The optimal cutoff value, 95% confidence interval, and area under the curve (AUC) were calculated using the DeLong method with the Youden’s index using the MedCalc Statistical Software Trial version (MedCalc Software bvba, Ostend, Belgium; http://www.medcalc.org; 2015). The risk factors affecting 24-hour mortality were investigated using univariate and multivariate logistic regression models. Statistical analyses were performed using Jamovi (version 1.6.13.0) and JASP (version 0.14.1.0) software. A p-value of less than 0.05 was considered statistically significant in all analyses.

## Results

In our study with 702 participants, the mean age of the patients was 68.8 ± 15.5 years, and 55% (n = 386) were male. Of the patients, 333 (47.4%) had hypertension (HT), 178 (25.4%) had chronic obstructive pulmonary disease (COPD), and 145 (20.7%) had coronary artery disease (CAD). The mean HCO_3_ level was 20.5 ± 8.3 mmol/L, mean lactate level was 6.7 ± 5.4 mmol/L, mean creatinine was 1.7 ± 1.6 mg/dL, mean potassium level was 4.8 ± 1.2 mmol/L, mean sodium level was 137.2 ± 7.4 mmol/L, mean PLT count was 250.0 ± 124.4 10^3^/μL, mean HS troponin I level was 471.4 ± 2,191.8 ng/L, mean urea level was 86.3 ± 72.4 mg/dL, mean PH was 7.2 ± 0.2, and mean Hgb was 79.9 ± 56.0 g/dL (Table 1).

**Table 1:** Descriptive statistics for patients’ sociodemographic, disease-related, and biochemical parameters. DM: diabetes mellitus; CAD: coronary artery disease; COPD: chronic obstructive pulmonary disease; CVA: cerebrovascular accident; CRF: chronic renal failure; HT: hypertension; PLT: platelet; Hgb: hemoglobin. Descriptive statistics are presented as mean ± standard deviation or median (minimum–maximum), depending on the distribution, while categorical variables are presented as numbers (%).

All Patients (n=702)	Mean ± SD/n (%)	Median (Min–Max)
Age	68.8 ± 15.5	70.0 (16.0–105.0)
Sex (%)		
Male	386 (55.0)	386 (55.0)
Female	316 (45.0)	316 (45.0)
DM (%)	108 (15.4)	
CAD (%)	145 (20.7)	
COPD (%)	178 (25.4)	
CVA (%)	129 (18.4)	
CRF (%)	84 (12.0)	
HT (%)	333 (47.4)	
Epilepsy (%)	59 (8.4)	
HCO₃ (mmol/L)	20.5 ± 8.3	20.0 (2.3–51.7)
Lactate (mmol/L)	6.7 ± 5.4	5.1 (0.0–30.0)
Creatinine (mg/dL)	1.7 ± 1.6	1.2 (0.2–21.8)
Potassium (mmol/L)	4.8 ± 1.2	4.6 (1.7–11.4)
Sodium (mmol/L)	137.2 ± 7.4	137.0 (105.0–189.0)
PLT (10^3^/μL)	250.0 ± 124.4	230.0 (1.0–1130.0)
HS troponin I (ng/L)	471.4 ± 2191.8	44.7 (1.2–27619.0)
Urea (mg/dl)	86.3 ± 72.4	61.5 (11.0–540.0)
PH	7.2 ± 0.2	7.2 (6.4–7.6)
Hgb (g/dl)	79.9 ± 56.0	96.0 (4.7–187.0)

When comparing patients based on their 24-hour mortality status, there was no significant difference in the mean age between survivors (69.3 ± 15.0 years) and non-survivors (67.4 ± 16.9 years) (p = 0.188); however, 95 (55.2%) men and 77 (44.8%) women died (p = 0.999). The rates of COPD, cerebrovascular accident (CVA), HT, and epilepsy were significantly higher in survivors than non-survivors (p = 0.001, p = 0.022, p = 0.021, and p = 0.012, respectively). There was no significant difference in terms of other comorbid conditions (p > 0.05 for each, Table 2).

**Table 2:** Twenty-four-hour mortality rates of the study patients according to sociodemographic characteristics, comorbid conditions, and laboratory findings. DM: diabetes mellitus; CAD: coronary artery disease; COPD: chronic obstructive pulmonary disease; CVA: cerebrovascular accident; CRF: chronic renal failure; HT: hypertension; PLT: platelet; Hgb: hemoglobin. Descriptive statistics are presented as mean ± standard deviation or median (minimum-maximum), depending on the distribution. Categorical variables are presented as numbers (%). *The t-test was used in independent groups. **The Mann–Whitney U test was used. ***Pearson’s chi-square test was used. The p-values indicated in bold are considered statistically significant (p<0.05).

	24-hour mortality		P-value
	Survivors (n = 530)	Non-survivors (n = 172)	
Age	69.3 ± 15.0	67.4 ± 16.9	0.188*
Gender (%)			
Male	291 (54.9)	95 (55.2)	0.999
Female	239 (45.1)	77 (44.8)	
Comorbid conditions			
DM (%)	87 (16.4)0	21 (12.2)	0.228
CAD (%)	111 (20.9)	34 (19.8)	0.824
COPD (%)	151 (28.5)	27 (15.7)	0.001
CVA (%)	108 (20.4)	21 (12.2)	0.022
CRF (%)	65 (12.3)	19 (11.0)	0.770
HT (%)	265 (50.0)	68 (39.5)	0.021
Epilepsy (%)	53 (10.0)	6 (3.5)	0.012
HCO₃ (mmol/L)	21.9 ± 8.3	16.2 ± 6.5	<0.001*
Lactate (mmol/L)	3.8 (0.0–27.0)	10.9 (1.0–30.0)	<0.001**
Creatinine (mg/dL)	1.2 (0.2–21.8)	1.4 (0.2–14.9)	<0.001**
Potassium (mmol/L)	4.7 ± 1.1	5.2 ± 1.4	<0.001*
Sodium (mmol/L)	137.0 ± 7.7	137.8 ± 6.6	0.142*
PLT (10^3^/μL)	244.0 (8.0–1130.0)	201.0 (1.0–509.0)	<0.001**
HS Troponin I (ng/L)	39.2 (1.7–27619.0)	63.5 (1.2–27302.0)	<0.001**
Urea (mg/dl)	61.5 (11.0–540.0)	61.5 (17.0–471.0)	0.813**
PH	7.2 ± 0.2	7.1 ± 0.2	<0.001*
Hgb (g/dl)	99.0 (4.7–187.0)	90.5 (6.2–183.0)	0.416**

The median/mean values of HCO_3_, PLT, and pH were significantly higher in survivors than in those who died within 24 h (p < 0.001 for each, Table 2). Furthermore, it was observed that the mean/median values of lactate, creatinine, potassium, and HS troponin I in patients who died within 24 hours were significantly higher compared to those who survived (p < 0.001 for each, Table 2). There were no significant differences in the other laboratory parameters based on the 24-hour mortality status (p > 0.05 for each).

**Figure 1:**
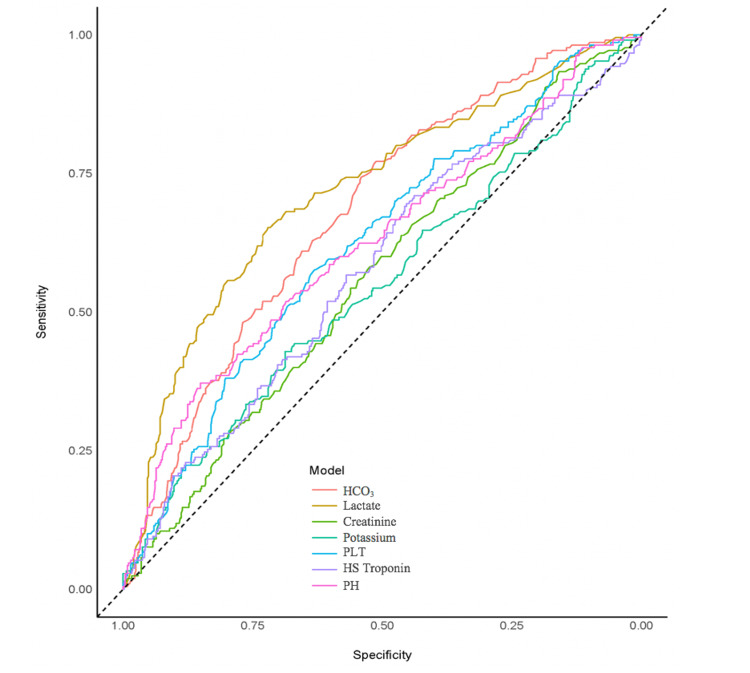
Regression curve for laboratory parameters in predicting one-month mortality.

The cutoff values for predicting 24-hour mortality were calculated as follows: ≤20.9 mmol/L for HCO_3_, >8.4 mmol/L for lactate, >1.8 mg/dl for creatinine, >5.05 mmol/L for potassium, ≤215 × 10^3^/μL for PLT, >32 ng/L for HS troponin I, and ≤7.157 for the pH (p < 0.05 for each, Table 3, Figure 1). The AUC, sensitivity, and specificity values calculated for each parameter are presented in Table 3.

**Table 3:** Calculation of cut-off values for laboratory parameters to predict 24-hour mortality using ROC curve analysis. AUC: area under the curve; CI: confidence interval.

24-hour mortality	AUC	Sensitivity	Specificity	Cut-off	%95 CI	p-value
HCO₃ (mmol/L)	0.710	80.81	54.15	≤20.9	0.675–0.743	<0.001
Lactate (mmol/L)	0.776	65.12	80.3	>8.4	0.743–0.806	<0.001
Creatinine (mg/dL)	0.592	71.51	45.66	>1.08	0.555–0.629	0.001
Potassium (mmol/L)	0.603	48.84	69.81	>5.05	0.566–0.639	0.001
PLT (10^3^/μL)	0.627	59.3	62.08	≤215	0.591–0.663	<0.001
HS Troponin I (ng/L)	0.610	73.84	45.85	>32	0.573–0.647	<0.001
PH	0.68	60.47	70	≤7.157	0.645–0.715	<0.001

Univariate and multivariate regression analyses were conducted for variables that showed significant differences between survivors and non-survivors. Accordingly, in the univariate model, the presence of COPD, CVA, HT, and epilepsy, along with HCO_3_, lactate, potassium, PLT, HS troponin I, and pH, were significant predictors of 24-hour mortality (each with p < 0.05). In the multivariate model, the presence of epilepsy, along with HCO_3_, lactate, potassium, and PLT values, were statistically significant (Table 4).

**Table 4:** The results of univariate and multivariate regression analyses regarding the predictive performance of significant laboratory parameters and comorbid conditions in predicting 24-hour mortality. COPD: chronic obstructive pulmonary disease; CVA: cerebrovascular accident; HT: hypertension; PLT: platelets; CI: confidence interval; OR: odds ratio. The p-values indicated in bold are considered statistically significant.

	Univariate model		Multivariate model	
24-hour mortality	OR (95% CI)	p-value	OR (95% CI)	p-value
COPD: present vs. absent	0.47 (0.30–0.73)	<0.001	0.79 (0.46–1.36)	0.398
CVA: present vs. absent	0.54 (0.33–0.90)	0.017	0.80 (0.45–1.45)	0.465
HT: present vs. absent	0.65 (0.46–0.93)	0.017	0.89 (0.58–1.36)	0.600
Epilepsy: present vs. absent	0.33 (0.14–0.77)	0.011	0.18 (0.07–0.52)	0.001
HCO₃ (mmol/L)	0.90 (0.88–0.93)	<0.001	0.96 (0.93–0.99)	0.047
Lactate (mmol/L)	1.19 (1.15–1.24)	<0.001	1.16 (1.09–1.23)	<0.001
Creatinine (mg/dL)	1.06 (0.96–1.18)	0.225	0.94 (0.81–1.09)	0.413
Potassium (mmol/L)	1.39 (1.21–1.60)	<0.001	1.21 (1.01–1.45)	0.041
Sodium (mmol/L)	1.02 (0.99–1.04)	0.174	1.01 (0.99–1.04)	0.299
PLT (10^3^/μL)	1.01 (0.99–1.01)	<0.001	1.01 (0.98–0.99)	<0.001
HS troponin I (ng/L)	1.01 (1-01–1-02)	0.025	1.01 (1.01–1.02)	0.122
pH	0.03 (0.01–0.08)	<0.001	1.97 (0.47–8.30)	0.358

## Discussion

Our study investigated parameters predicting mortality in patients who underwent rapid sequence intubation in the emergency department. While these parameters were previously studied for specific conditions, there was no comprehensive study in the relevant literature to the best of our knowledge. In this study, the presence of COPD, CVA, HT, and epilepsy, as well as the values of HCO_3_, lactate, potassium, PLT, HS troponin I, and pH, significantly predicted 24-hour mortality in univariate analysis. Additionally, in the multivariate model, the presence of epilepsy, along with HCO_3_, lactate, potassium, and PLT values, emerged as significant predictors of 24-hour mortality.

The lactate value in blood gas analysis is considered an important parameter and is recognized as a significant biomarker for assessing the hemodynamic status of the patient. Lactate itself is not toxic, but an increase in its concentration indicates significant changes in homeostasis. Therefore, a number of previous studies explored its potential as a predictive parameter for mortality [13,14]. A study by Swan et al. in the emergency intensive care unit reported an association between elevated lactate levels and mortality [15]. Similarly, in a multicenter study involving patients with septic shock, the implementation of a lactate-targeted strategy resulted in a substantial decrease in mortality [16]. Lactate clearance upon patient admission may indicate a reduction in global tissue hypoxia and is associated with decreased mortality rates [17]. In the present study, the median lactate value was lower among survivors (3.9 mmol/L) compared to non-survivors (9.2 mmol/L). Consistent with other studies in the literature, these values in the present study emerged as significant predictors of 24-hour mortality in the multivariate model. Blood gas analysis and lactate measurement enable the early recognition of the risk for fatal outcomes and are cost-effective methods [14,18].

In the present study, the median pH level in arterial blood gas analysis was 7.2. Similarly, Duarte et al. reported the median pH value in intubated patients as 7.28 [19]. Kraut and Madias investigated the treatment of metabolic acidosis and suggested that acidosis was associated with increased mortality by potentially facilitating cardiac arrhythmias and suppressing immune responses [20]. In the present study, the mean pH level was significantly higher in survivors compared to non-survivors, as anticipated. In a study by Siu et al., the median HCO_3_ value was 23 mmol/L in intubated patients. In the present study, the mean HCO_3_ value was 21.9 ± 8.3 mmol/L in survivors, while it was 16.2 ± 6.5 mmol/L in non-survivors [21] and HCO_3_ significantly predicted 24-hour mortality. Unlike our study, this parameter was not identified as a significant predictor for mortality in a study conducted by Zheng et al. However, this outcome might be expected considering that the patients with acute kidney failure were included in that study group [22]. A study, which investigated bicarbonate and mortality in patients with chronic renal failure, reported that low bicarbonate values increased the risk of death by 2.6 times but did not increase mortality in patients without chronic renal failure [23].

Potassium concentration abnormalities are associated with morbidity and mortality. In recent years, there has been growing consideration that even minor fluctuations within the normal range of serum potassium concentrations may be associated with mortality [24]. In the present study, the mean potassium level was 5.0 ± 1.4 mmol/L in non-survivors, and potassium levels were identified as a significant predictor of 24-hour mortality in the multivariate model. It is known that thrombocytopenia accompanies fatal conditions, including sepsis and disseminated intravascular coagulation. Previous studies reported that thrombocytopenia was directly associated with mortality, consistent with the results of the present study [25].

In a study conducted by Daniel et al., cardiac markers were examined in patients without suspected coronary syndrome, and it was found that cardiac markers were significantly elevated in patients who underwent intubation [26]. Another study with over 250,000 patients investigated the effects of elevated troponin levels in a large sample of patients who underwent troponin testing for any clinical reason. It was reported that even slightly elevated troponin levels, above the normal range, were associated with mortality [27]. Similarly, in a study conducted by Jia et al., the elevated HS troponin I levels were directly associated with mortality [28]. In the present study, the median HS troponin I value was 39.2 ng/L in survivors and 63.5 ng/L in non-survivors. Although this parameter has been associated with mortality in the literature, it was not identified as a significant predictor of 24-hours in the multivariate model in the present study.

The retrospective design of the present study is the most significant limitation. As a result, we experienced challenges in accessing complete data and could not access the treatment protocols administered to patients. Furthermore, the single-center design of our study and the relatively smaller sample size were associated with challenges for generalizing the findings. Another limitation is the absence of follow-up arterial blood gas analysis. The prognosis of the patient may be affected by the time taken for rapid sequence intubation, a factor dependent on the individual administering the procedure. The lack of standardized protocols for this procedure introduces a limitation in generalizing the findings of the study.

## Conclusions

This study highlights the significance of certain laboratory parameters, particularly arterial blood gas values, in predicting 24-hour mortality among patients undergoing rapid sequence intubation in the emergency department. Specifically, our findings suggest that elevated lactate, potassium, and HS troponin I levels, alongside decreased HCO_3_, PLT, and pH values, are strong indicators of increased mortality risk within this critical time frame. The results demonstrate that the early recognition of these markers allows emergency physicians to assess the severity of a patient's condition promptly, potentially guiding clinical decisions to improve outcomes.

Future multicenter and prospective studies are essential to validate these findings and to develop standardized protocols for rapid sequence intubation and patient monitoring. Such efforts would not only enhance the predictive power of laboratory parameters but also contribute to the optimization of patient care in emergency settings.
